# Supplementary figures and images for: Combining cash transfers and cognitive behavioral therapy to reduce antisocial behavior in young men: A mediation analysis of a randomized controlled trial in Liberia
Source: PLoS One. 2023 Mar 17;18(3):e0273891. doi: 10.1371/journal.pone.0273891 (PMC10022758; doi:10.1371/journal.pone.0273891)

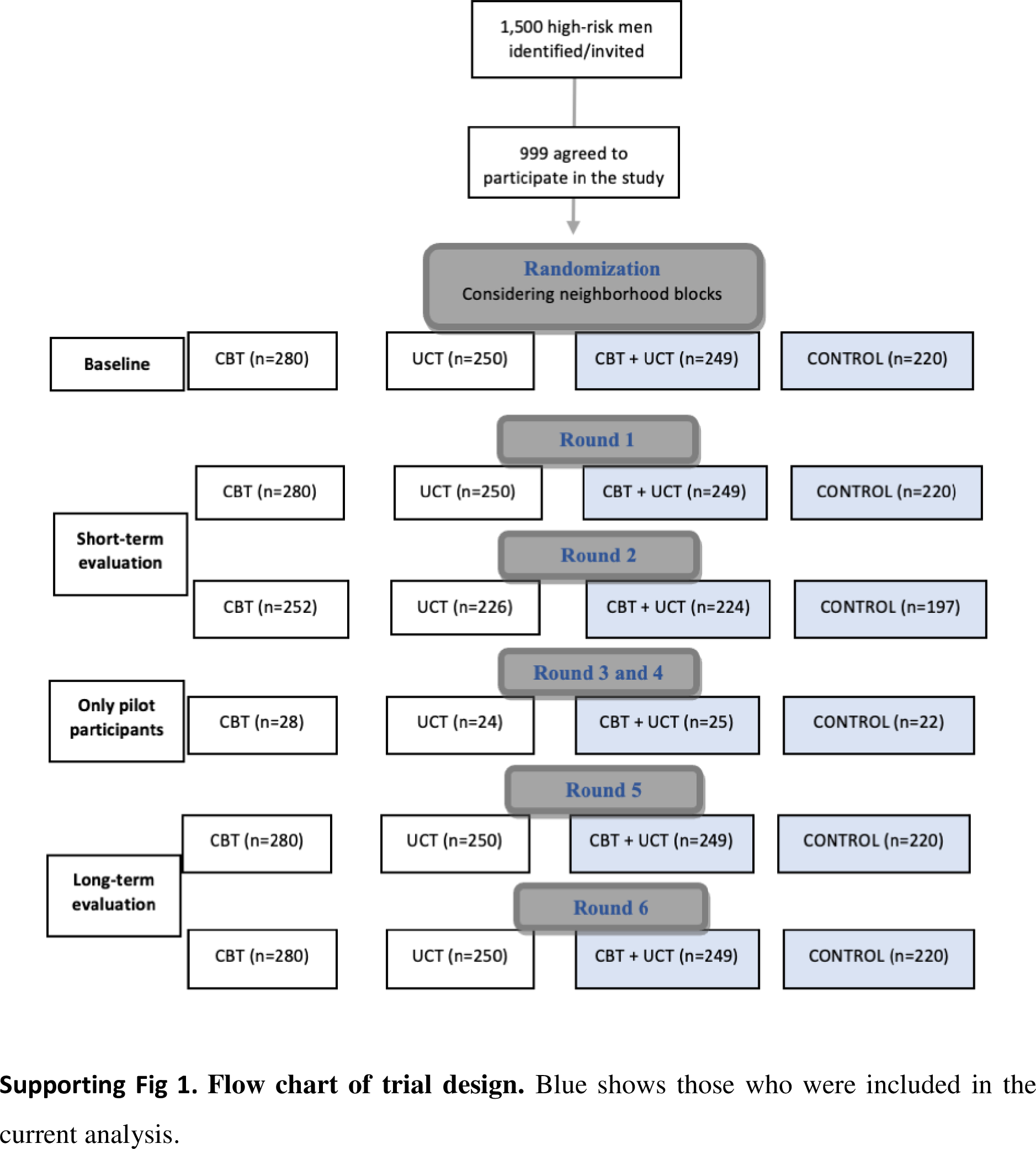

Supplement: S1 Fig — Blue shows those who were included in the current analysis. (TIF) [file pone.0273891.s001.tif]
